# Clinical hints to diagnosis of attenuated forms of Mucopolysaccharidoses

**DOI:** 10.1186/s13052-018-0551-4

**Published:** 2018-11-16

**Authors:** Miriam Rigoldi, Elena Verrecchia, Raffaele Manna, Maria Teresa Mascia

**Affiliations:** 1 Centro Malattie Rare, ASST-Monza, Ospedale San Gerardo, Via Pergolesi, 33 20900 Monza, MB Italy; 2 Centro delle febbri periodiche e malattie rare, Policlinico Gemelli, Università Cattolica Roma, Rome, Italy; 3 Patologie dell’apparato locomotore a genesi immunologica, Università di Modena e Reggio Emilia, Azienda Ospedaliero-Universitaria, Policlinico di Modena, Modena, Italy

**Keywords:** Mucopolysaccharidosis, Attenuated MPS, Musculo-skeletal signs, Heart valve disease, Eye involvement

## Abstract

The mucopolysaccharidoses (MPS) are clinically similar but also heterogeneous in terms of major or minor involvement of different organs/systems, burden of disease, and rate of progression. The attenuated forms of MPS, due to their less severe presentations, are more difficult to diagnose and often receive a significantly delayed diagnosis. On the other hand, the diagnosis is very important since the attenuated forms may benefit from earlier treatments. The aim of this paper is to describe the natural history and the clinical signs useful to arise a suspicion of an attenuated form of MPS. MPS patients usually show a cluster of signs and symptoms, one of which may be the trigger for an evaluation by a specialist. Individuals with attenuated MPS are mostly cognitively normal, and dysmorphisms of the facies may be mild or absent. The most frequently involved organs/systems are the osteoarticular system, heart, and eyes. These patients may also have hepatosplenomegaly, hearing loss, and respiratory problems. When they are referred to a specialist (rheumatologist, cardiologist, ophthalmologist, surgeon, orthopedist, etc.) for their main complaint, the other signs and symptoms are likely to be missed in the medical history. To avoid missing data and to save time, we propose a semistructured medical history form to be filled in by the patients or their caregivers while waiting for evaluation by a specialist.

## Background

Mucopolysaccharidoses (MPS) are genetic, multisystem, and progressive diseases where virtually all the organs and systems are involved. Thus far, seven types of MPS are known: type I, II, III, IV, VI, VII, and IX. These are similar, but also have a significant inter- and intra-disease variability in their onset, organs and systems involvement, and clinical course.

The intra-disease variability is mainly due to different underlying mutations and the consequent degree of residual enzyme activity [1]; usually there is a correlation between severity of the disease and the age of its onset, with the more severe disease having an earlier onset. The attenuated forms of MPS are more difficult to diagnose since the disease progresses silently over decades and early symptoms are subtle and may be overlooked by physicians who are not familiar with the disease. The more frequent signs and symptoms of late-onset MPS patients are related to heart valve, joints, and eyes (Table 1) [2, 3]. A frequent picture is that of a patient with the onset of noninflammatory joint contractures in childhood which are initially diagnosed as rheumatoid arthritis, juvenile idiopathic arthritis, or scleroderma and, after many years of misdiagnosis, these are identified as MPS signs [4]. In a number of cases, joint stiffness is associated with a heart murmur and/or eye problems [2]. The accumulation of glycosaminoglycan (GAGs) in the lysosomes is the pathogenic trigger of MPS manifestations. Since nowadays the majority of MPS are treatable disorders and the attenuated forms can benefit consistently from early treatments, our efforts should focus on shortening the gap between the onset of symptoms and the final diagnosis.

**Table 1:** Skeletal, cardiac, and ocular features for each type of attenuated MPS as summarized from the literature +++ = most/all; ++ = several; + = few

Clinical features	MPS I	MPS II	MPS III	MPS IV	MPS VI	MPS VII	MPS IX	References
Skeletal								[2–4, 6–12, 24, 25, 28–30, 32, 33]
Short stature	+	++	+	+++	++	++	+	
Gibbus deformity	+	++	–	–	++	++	–	
Valgus knee	+	++	–	+++	++	++	–	
Pectus carinatum	+	+	–	+++	+	+	–	
Scoliosis	++	++	+	+++	++	+	–	
Hip dysplasia	+++	++	+	+++	++	+++	–	
Atlanto-axial instability	+	–	–	+++	++	+	–	
Cord compression	++	++	–	+++	++	++	–	
Carpal tunnel syndrome	+++	+++	–	–	+++	+	–	
Arthropathy	+++	+++	+	+++	+++	+++	+++	
Joint stiffness	+++	+++	+	–	+++	+++	–	
Joint laxity	–	–	–	+++	–	–	–	
Joint effusion	–	–	–	–	–	–	+++	
Heart								[14, 16, 17, 19]
Valvulopathy	+++	+++	+	+	+++	++	UN	
Hypertrophic cardiomyopathy	+	++	–	–	+	+	UN	
Eye								[21, 22]
Corneal clouding	+++	+	+	+	+++	+++	UN	
Retinal degeneration	+++	++	++	++	UN	UN	UN	
Glaucoma	++	+	+	+	++	++	UN	

+++ most/all, ^++^ several, ^+^ few

*MPS* mucopolysaccharidoses, *UN* unknown

The aim of this paper is to describe the natural history and the most common signs useful to reach the suspicion of an attenuated form of MPS.

### What is relevant for suspicion?

The presence of signs and symptoms in a cluster is very important for a suspicion of MPS.

This paradigm might not be easily taken into account because a specialist may only look at signs within her/his specific competence, without being able to look into the other, possibly milder, signs or symptoms that should arouse a clinical suspicion.

Clinical history is essential since an attenuated patient can meet a specialist for a specific problem but, during the medical history interview, another two or three mild signs or symptoms may be noticed which can lead to suspicion of MPS. The family history should also be collected since MPS are hereditary disorders and other family members could be affected.

With this purpose in mind, we are proposing a semistructured medical history form (see Appendix) that can be used under different settings by rheumatologists, cardiologists, ophthalmologists, surgeons, and other specialists, and applies to all of the patients who present one of the typical somatic signs of MPS. This is adapted from a previous questionnaire proposed for rheumatologists [5], and it will be useful to raise a suspicion for all MPS types, except for MPS type III which has mainly neurocognitive signs.

In summary, symptoms present in a cluster, and family and individual histories are of upmost importance.

### The natural history in attenuated forms of MPS

For each type of MPS two different phenotypes can be recognized, severe and attenuated. Such a classification is sometimes useful, although there is a phenotypic continuum among the different types.

The two main characteristics of all the attenuated types of MPS are the later onset and the slower progression compared with the severe forms. The disease onset is generally in the pediatric age, but the signs are mild and for this reason they can be overlooked and/or misdiagnosed.

The height of the adult individual is usually lower than that of the general population, but in some cases patients may show normal growth [2, 6].

With the exception of attenuated MPS III, where the first sign is cognitive/behavioral involvement, in the other attenuated forms of MPS the first signs are in general somatic and the majority of patients show normal intellectual performances with only a small number of Hurler-Scheie and attenuated Hunter patients showing variable degrees of learning and attention difficulties [2, 7].

For these, osteoarticular manifestations are the first or the second sign of disease expression appearing in MPS I Scheie (the attenuated form of MPS I) at a median age of 7.5 years in around 70% of patients [8] and this is preceded by the occurrence of umbilical/inguinal hernias at a median age of 4.6 years [8–10]. Heart valve disease and corneal clouding are also signs found in around 70% of MPS I Scheie patients, appearing at the end of the first decade of life.

**Fig. 1:**
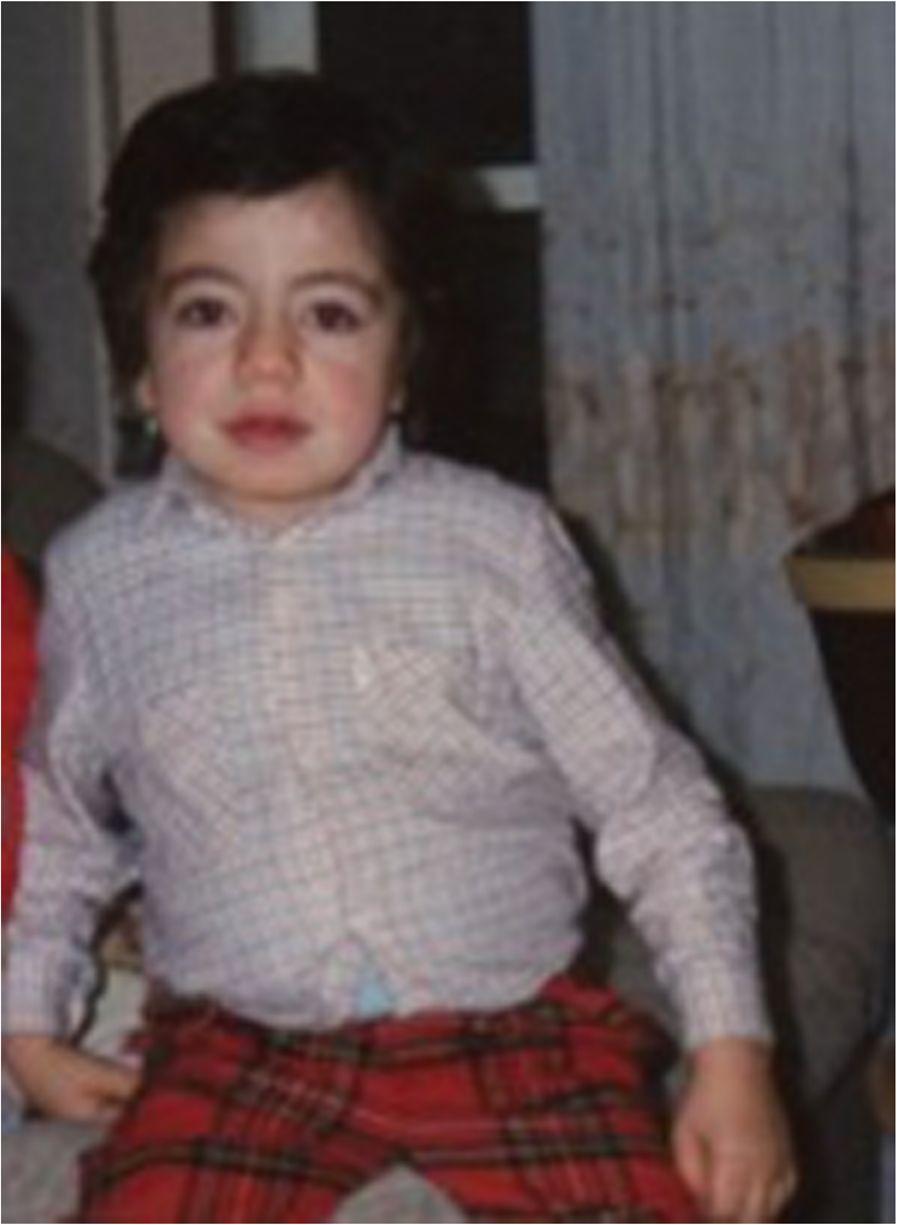
Six-year-old MPS II attenuated patient with very mild facial signs of the disease

The attenuated forms of MPS may have mild facial dysmorphisms which begin to appear in childhood and tend to worsen over time (Fig. 1) [8, 9]. Dysmorphisms, however, are not the rule since in many attenuated patients they are very mild or absent [11].

Life expectancy can be nearly normal, and some patients may also reproduce [12, 13]. In summary, there is slow progression, no cognitive delay, and hernias, osteoarticular involvement, eye, and heart valve disease are the most frequent signs.

### Which kind of cardiac involvement is typical of MPS?

Cardiac involvement is a main sign, common to all the attenuated forms of MPS including MPS III, and heart malfunctioning is one of the causes of major somatic complications and death [14] (see also Boffi et al. [15] in this supplement**)**.

Valvulopathy is the main cardiac sign in MPS individuals with slowly progressing disease. The mitral and aortic valves are the most commonly affected, irrespective of the type of MPS, with valve thickening and regurgitation, followed by stenosis [16, 17].

MPS I, II, and VI patients, whose GAG accumulation consists of dermatan and heparan sulfate, have a faster progression of valvulopathy compared with MPS III and IV patients where dermatan sulfate is not stored [16, 18].

Other common features are left ventricular hypertrophy, abnormal diastolic function, and coronary artery disease. Pulmonary hypertension may arise as a consequence of airways obstruction, deformity of the ribs, or GAG storage in the walls of pulmonary vessels. The GAG storage also causes an abnormal conduction of the electric signal (arrhythmia, complete heart block) [18, 19]. Enzyme replacement therapy (ERT) may have a positive impact on left ventricular hypertrophy [19, 20] while it generally does not improve the cardiac valve disease, although it may attenuate or delay its progression [19, 20].

Cardio-surgery is a good option to treat these complications but, as with other surgical procedures, the risk of adverse events during or immediately after surgery increases in MPS patients [17] (see Moretto et al. in this Supplement [18]). Rehabilitation may be difficult in the presence of orthopedic issues. In summary, valvulopathy is the most frequent cardiac sign in attenuated MPS.

### Which kind of ocular involvement is typical of MPS?

**Fig. 2:**
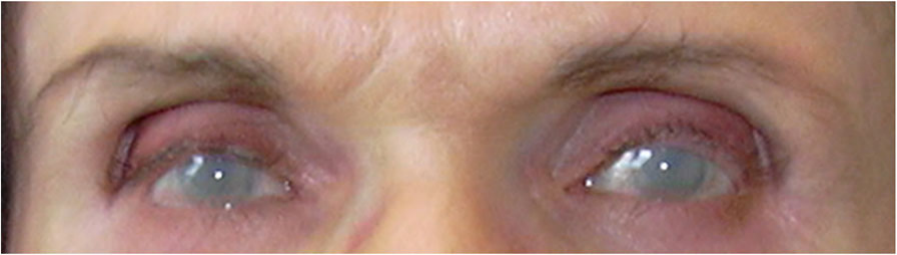
Severe corneal clouding in a 60-year-old patient affected by MPS IVA

Corneal clouding (Fig. 2) is another typical finding of attenuated MPS. It progresses over time and often requires corneal transplantation in early adulthood [21, 22] (see also Del Longo et al. in this Supplement [23]). This might be effective in ameliorating the sight of MPS patients provided they are not affected by retinopathy. Corneal clouding is frequently severe in MPS I and VI patients, while it is less severe in MPS IV and MPS VII and usually mild in MPS II and III. In MPS I Scheie, the onset of corneal clouding was reported at a median age of 10.5 years and it can be associated with glaucoma, increased intraocular pressure, and retinal degeneration [8, 9, 21, 22]. Retinal degeneration is frequently seen in adult patients affected by MPS I, II, and III, more rarely in MPS IV patients, and it has not been reported for MPS VI patients [6, 21–24]. Ocular manifestations in MPS IX have not been studied [22]. In summary, corneal clouding and retinal degeneration are associated with attenuated MPS.

### Which kinds of osteoarticular manifestations are typical of MPS?

**Fig. 3:**
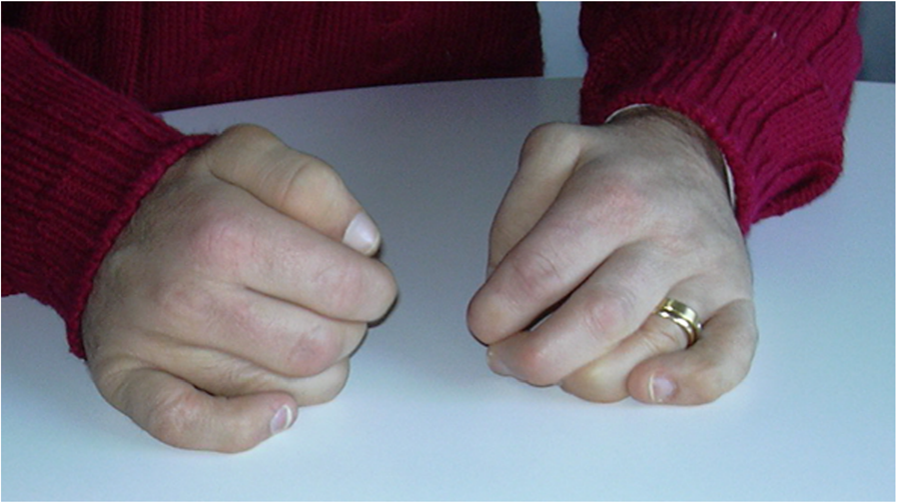
Claw hands of an adult patient affected with attenuated MPS II

**Fig. 4:**
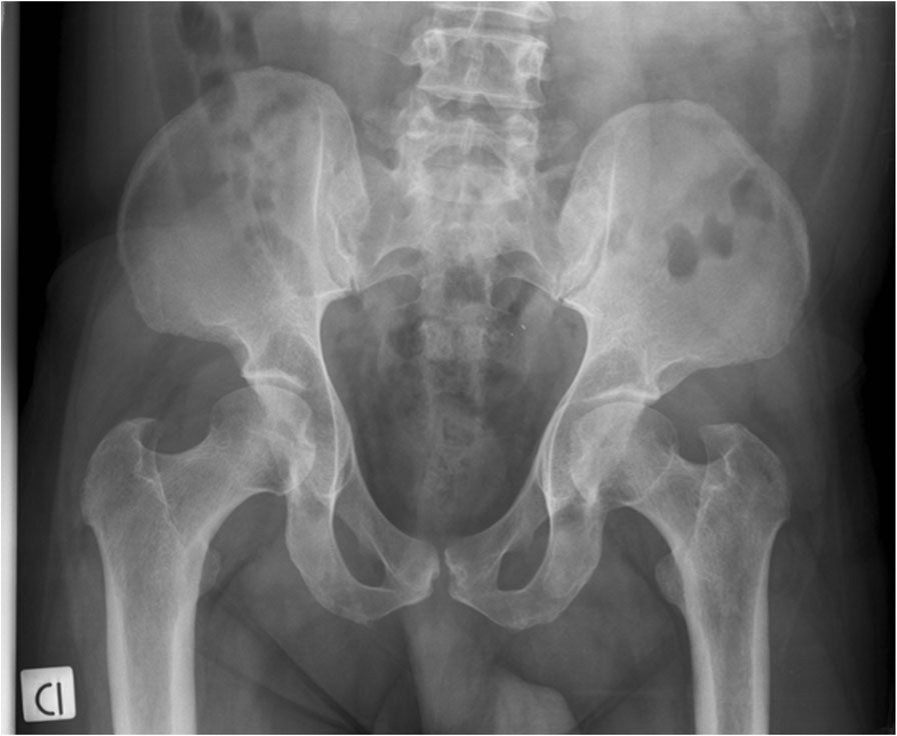
Bilateral coxarthrosis with reduction of the acetabular spaces in a patient affected by MPS I Scheie

The GAG deposition in the growth plate causes endochondral ossification of the physis. Thus, it results in growth decline and eventually in short stature [25–28]. The storage is always evident in the cartilage, tendons, and joints. Studies on MPS animal models have documented an abnormal composition of the cartilage matrix and apoptosis of chondrocytes with release of nitric oxide and elevated levels of tumor necrosis factor (TNF)-alpha. The inflammation plays a pivotal role inside the joints, but it is not clinically evident [29]. Features are very similar to those of human osteoarthritis, although the presence of high levels of TNF-alpha in the synovium may also explain why the clinical picture can mimic rheumatic inflammatory diseases [30]. Over time, patients with milder forms of MPS may develop clinical pictures characterized by joint contractures with reduced range of motion, claw hands (Fig. 3), trigger finger, carpal tunnel syndrome (CTS), dysostosis multiplex with atlanto-axial instability associated with hypoplasia of the odontoid process, kyphoscoliosis, hip dysplasia, varus knee, pes cavus/planus, and genu valgum. The x-ray images may also be not typical of MPS, showing aspecific osteoarthrosis in certain cases (Fig. 4). Patients with all MPS types (except MPS III) can suffer from compressive myelopathy both in the cranio-cervical and dorso-lumbar regions, with severe neurological consequences [6, 8–11, 24, 31, 32].

Spinal canal stenosis is caused by thickening of the soft elements (dura, ligamentum flavum, cruciate ligaments), disc bulging, spinal deformities, or a combination of these factors [33].

MPS IVA can usually be clinically distinguished from other MPS by the presence of additional skeletal manifestations derived from a unique epiphyseal chondrodysplasia and laxity of joints [6, 32].

Stiffness and contractures usually start in childhood; they are not associated with swelling, warmth, or tenderness, and are especially localized at the hands (Fig. 3). The pathological accumulation in the tendons may involve interphalangeal joints and causes important impairment to hand function (claw hands). These aspects can also mimic camptodactyly or diabetic cheiroarthropathy.

For all the above reported presenting signs, patients will come to the attention of the rheumatologists and/or orthopedic surgeons or neurosurgeons.

The medical literature contains individual case reports or small cohorts of attenuated MPS patients with onset of symptoms in childhood who were finally identified after many years and sometimes decades, with a range up to 50 years. In some of these patients, misdiagnoses of rheumatoid arthritis, juvenile idiopathic arthritis, or scleroderma had been maintained for several years [8, 10, 34].

In the very few reports on MPS IX, patients are described with periarticular nodular soft tissue masses with short stature, episodes of painful joint swelling, no joint stiffness and no kypho-scoliosis [35].

Another important orthopedic aspect is the occurrence of carpal tunnel syndrome (CTS) in children or adolescents, which is otherwise rare in the pediatric age. Thus, a history of juvenile carpal tunnel release surgery must raise the suspicion of a metabolic or genetic disorder and especially of MPS (I, II, and VI) which accounts for more than half of the cases in this age group [8]. In MPS, clinical signs typical of adult CTS such as nocturnal paresthesia may be absent; the most frequent finding is a difficulty with fine motor tasks [33]. In summary, joint contractures without inflammatory signs, carpal tunnel syndrome, and radiological signs of (attenuated) dysostosis multiplex are particularly suggestive of MPS.

### Are other organs involved, and how?

All MPS types show recurrent infections of the upper respiratory tract, such as recurrent rhino- and oto-sinusitis (especially in the first decade of life), restrictive lung disease, and obstructive sleep disturbances/snoring [32, 36, 37].

The attenuated forms may present with airway obstructions from the rhino-pharynx to the peripheral bronchi. The submucosal storage material is often the cause of the upper airway obstruction.

A complication, particularly frequent and often lethal in MPS II and IV, is tracheal stenosis caused by tracheomalacia and often associated with external compression. Usually, these stenoses arise in the teenage years and are progressive; thus far, no conclusive guidelines have been set regarding their management and treatment, although surgical treatment have been successful in some patients [38, 39].

Hypoacusia is frequent and may be due to conductive or sensorineural hearing loss, or both. Hearing loss may develop in attenuated MPS, most commonly in the high frequency range, and probably due to accumulation of GAGs both in the ossicles of the middle ear and in the acoustic nerve [40].

Gastrointestinal involvement is also described as hepatosplenomegaly or diarrhea to a variable degree and percentage. Hernias (inguinal, umbilical) are present in approximately 65% of MPS I Scheie and they are often the onset sign [41]. In summary, sleep disturbances, tracheomalacia and/or stenosis, hypoacusia hernias, and mild hepatosplenomegaly are often associated with the clinical picture of attenuated MPS.

### Is apparently isolated cognitive insufficiency a sign of MPS; the MPS III attenuated phenotype

MPS III differs from the other types of MPS in that it is mainly a neurodegenerative disorder with a milder somatic involvement. MPS III is characterized, after an initial symptom-free interval, by behavioral problems followed by progressive mental deterioration leading to severe dementia [42]. If one considers the spectrum of the classic phenotypes, subtype A is in general more severe and has an earlier age of death than the other subtypes [43–45].

As for the other types of MPS, MPS III patients can also be divided into two phenotypic groups, rapidly progressing and slowly progressing, according to the progression of their clinical course [46]. Among the slowly progressing, there are patients who can reach remarkably advanced ages with a clinical picture characterized by unexplained mental retardation/insufficiency associated with behavioral problems such as restlessness, screaming, hypersensitivity to touch, anxiety, aggression, and sleep disturbances, including frequent awaking. The neurological degeneration arises in the fourth to sixth decades with loss of motor function, loss of speech, epilepsy, loss of sphincter control and, in the end, swallowing problems [47].

In this attenuated MPS III phenotype, the somatic involvement could be very mild and present only in advanced age. The most frequent physical problems consist of cardiac disease (cardiomyopathy, arrythmias), arthritis, skin blistering, hernias, and susceptibility to infections.

For all these reasons, unexplained mental retardation and behavioral problems in adults should prompt metabolic investigation for MPS III [47, 48]. In summary, MPS III must be suspected in patients with unexplained cognitive delay mainly if associated with behavioral problems.

### What are the red flags that should prompt the diagnosis of an MPS?

A group of rheumatologists and pediatricians together with MPS experts have developed a diagnostic algorithm based on rheumatologic aspects of MPS [34].

The suspicion of MPS should arise when a patient shows important joint contractures (not associated with either swelling or local inflammation) or stiffness. Moreover, inflammatory markers are absent, there is no response to steroids or nonsteroidal anti-inflammatory drugs, and no radiological evidence of erosive aspects. Urinary GAG might be absent in adult patients with attenuated forms of MPS.

For this reason, the diagnosis should not be excluded if the first quantitative GAG dosage is negative. All the symptoms of the patients should be re-evaluated (Table 2) and other tests (qualitative urinary GAG, enzyme, and molecular analysis), (see Filocamo et al. in this Supplement [49]) performed in case the suspicion is confirmed.

**Table 2:** “Red flags” signs for early diagnosis of attenuated mucopolysaccharidoses

“Red flag” sign	
Noninflammatory joint stiffness	+++
Juvenile Carpal tunnel syndrome	+++
Heart valve disease	+++
Corneal clouding	+++
Skeletal abnormalities	++
Hernias	++
Hepatomegaly	+

Produced from data in [2–4, 6–12, 14, 16, 17, 19, 21, 22, 24, 28–30, 32, 33]

+++ very suggestive, ++ suggestive, + faintly suggestive

A semistructured form to be self-completed by the patients or their caregivers while waiting for the evaluation of a specialist (mainly cardiologists, rheumatologists, orthopedists, and ophthalmologists) is reported in Appendix. It has been retrospectively tested on 16 clinical records of attenuated MPS; if this questionnaire had been used, all the patients could have been diagnosed earlier. The advantage of this semistructured history is that the physician has a selective medical history already available at hand for excluding or suspecting an MPS or other rare genetic diseases involving the bones.

## Conclusions

The attenuated forms of mucopolysaccharidoses represent a continuum from the most severely affected patients showing different degrees of severity. They may remain apparently asymptomatic for years with a silently progressing disease and only be recognized at an advanced disease stage when it is too late for any intervention.

Awareness among medical specialists in adult medicine should be raised so that they can play an important role not only in the management but also in the diagnosis of MPS.

A precise and sophisticated diagnosis of a particular type of MPS is not expected only on clinical grounds since there is a significant overlap between the various types. It will be enough for the specialist to suspect that the patient may suffer from MPS.

In this respect, signs such as umbilical and/or inguinal hernia, juvenile CTS, bone deformities and joint contractures without apparent inflammation, heart valve disease, or eye problems should be regarded as red flags useful to raise a clinical suspicion (Table 2). An accurate medical history is essential to identify all these signs. Our tool for a semistructured medical history will help to refer the patient to the appropriate physician (MPS expert) to avoid a medical odyssey and start the appropriate treatment as soon as possible. MPS are treatable disorders, and their diagnosis has implications for the patient’s quality of life and life expectancy.

## Appendix

**Table 3:** Semi-structured history form to be completed by the patient while waiting for the specialist evaluation (cardiologist, rheumatologist, orthopedist, ophthalmologist)

	Questions	Answers			I don’t understand	Observations
		Yes	No	I don’t know		
Eye involvement						
Have you ever had ocular problems?						
*If yes*	Has an eye care specialist ever said that you suffer from:					
	• corneal opacities					
	• retinal pigmentation					
	• retinal degeneration					
	• glaucoma					
	• atrophy of the optic nerve					
Heart involvement						
Have you ever had heart problems?						
*If yes*	Has a cardiologist ever told you that you have heart valve abnormalities?					
Ear, nose and throat involvement						
Have you ever had hearing and /or respiratory problems?						
*If yes*	Have you ever had:					
	• permanent rhinitis					
	• repeated infections of the upper airways					
	• hearing loss					
	• language delay					
	• laryngotracheal stenosis					
	• tonsillectomy					
	• adenoidectomy					
	• wheezing with your shortness of breath					
	• sleep apnea					
Musculoskeletal involvement						
Have you ever had musculoskeletal problems?						
*If yes*	Have you ever had:					
	• spinal cord compression (a severe thickening of the tissue along the spine)					
	• contractures or joint deformity • skeletal deformity not secondary to trauma					
	• fixed flexion deformity of fingers					
	• carpal tunnel syndrome					
	• pain in or around your joints					
	• orthopedic surgery (Please list any surgical procedures you have undergone)					
	• valgus knee, trigger finger, joint laxity					
Abdominal involvement						
Have you ever had abdominal problems?						
*If yes*	Have you ever had:					
	• an enlarged liver (hepatomegaly)					
	• An enlarged spleen (splenomegaly)					
	• an umbilical hernia					
	• an inguinal hernia					
Family history						
	Are your parents blood relatives?					
	Did you have a slower growth during early childhood?					
	Were you born prematurely?					
	Do you think that there are any conditions or illnesses which run in your family? If do, please specify which ones:					

## Abbreviations

CTS: Carpal tunnel syndrome
ERT: Enzyme replacement therapy
GAG: Glycosaminoglycan
MPS: Mucopolysaccharidosi(e)s
TNF: Tumor necrosis factor
